# Performance of PRP Associated with Porous Chitosan as a Composite Scaffold for Regenerative Medicine

**DOI:** 10.1155/2015/396131

**Published:** 2015-03-02

**Authors:** Andréa Arruda Martins Shimojo, Amanda Gomes Marcelino Perez, Sofia Elisa Moraga Galdames, Isabela Cambraia de Souza Brissac, Maria Helena Andrade Santana

**Affiliations:** Department of Engineering of Materials and of Bioprocesses, School of Chemical Engineering, UNICAMP, P.O. Box 6066, 13083-970 Campinas, SP, Brazil

## Abstract

This study aimed to evaluate the *in vitro* performance of activated platelet-rich plasma associated with porous sponges of chitosan as a composite scaffold for proliferation and osteogenic differentiation of human adipose tissue-derived mesenchymal stem cells. The sponges were prepared by controlled freezing (−20, −80, or −196°C) and lyophilization of chitosan solutions (1, 2, or 3% w/v). The platelet-rich plasma was obtained from controlled centrifugation of whole blood and activated with calcium and autologous serum. The composite scaffolds were prepared by embedding the sponges with the activated platelet-rich plasma. The results showed the performance of the scaffolds was superior to that of activated platelet-rich plasma alone, in terms of delaying the release of growth factors and increased proliferation of the stem cells. The best preparation conditions of chitosan composite scaffolds that coordinated the physicochemical and mechanical properties and cell proliferation were 3% (w/v) chitosan and a −20°C freezing temperature, while −196°C favored osteogenic differentiation. Although the composite scaffolds are promising for regenerative medicine, the structures require stabilization to prevent the collapse observed after five days.

## 1. Introduction

In tissue engineering, a scaffold is a three-dimensional matrix for the stimulation of cell proliferation and the formation of new tissue. To achieve this goal, scaffolds must meet some specific requirements such as mimicking the native extracellular matrix (ECM) of the target tissue; allowing cell attachment, migration, proliferation, and differentiation to and maintenance of the target phenotype; promoting vascularization and nutrient delivery; and having a biodegradation rate and mechanical properties that are adequate to support the formation of the new tissue [1, 2].

According to Crane and Everts, tissue regeneration is based on a proliferation triangle composed of the three fundamental elements of cells, growth factors (GFs), and scaffolds, which are in a close relationship through their capabilities. Thus, scaffolds provide the conductive matrix for supporting the genic capability of progenitor cells mediated by the inductive capability of GFs [3]. Optimal conditions for tissue regeneration must come from the interaction of the properties of those elements.

The cell responses to the surface chemistry of scaffolds depend on their hydrophobicity, protein adsorption, surface charge, and roughness, softness, and stiffness. Additionally, the porous architecture characterized by the pore size, porosity, connectivity, and tortuosity plays important roles, as described and discussed by Chang and Wang [4]. Therefore, bringing together all these properties in solid scaffolds is an enormous technological challenge.

The fibrin matrix is the natural scaffold formed from the coagulation cascade in the healing process. Under injury, thrombin cleaves the soluble plasma protein fibrinogen into peptide fragments, yielding insoluble fibrin peptides that aggregate and form fibrils. A fibrin network is then formed, which entraps platelets and other blood components [5, 6]. Calcium acts as a cofactor of thrombin, modulates the elongation of fibers during polymerization by promoting lateral branching, and functions in clot stability [7, 8]. Calcium and thrombin also activate platelets, allowing the release of GFs and cytokines. Therefore, the fibrin matrix provides an optimized medium for cell proliferation and healing.

Based on these features, platelet-rich plasma (PRP) has been widely used in regenerative medicine. PRP is an autologous product prepared from whole blood by separating the red blood cells and concentrating the platelets and other components of plasma. However, although viscoelastic, the fibrin matrix alone lacks stability to be efficient when regenerative medicine is the goal.

To mimic the natural healing process and improve the stability of the fibrin matrix, we propose in this work the use of the fibrin network from activated PRP with porous chitosan as a composite scaffold for tissue regeneration.

Our hypothesis is that the chemical nature of the chitosan surface supports the fibrin network by electrostatic attachment, thus prolonging its stability, without changing the paracrine affinity of mesenchymal cells to fibrin fibers. As a consequence, the composite scaffold must improve cell proliferation and tissue differentiation compared to PRP alone.

Related works showed the effects of chitosan on blood coagulation through the strong adhesion of platelets to the surface of chitosan particles, as well as chitosan (0.1–1 mg/mL) incorporated with PRP to enhance the release of PDGF-AB and TGF-*β*1 from platelets [9, 10]. Shen et al. demonstrated that chitosan could be an appropriate substitute for thrombin, as an agonist in PRP preparation [11]. Chang et al. showed that growth factors could sustain release until 12 h at approximately 1 ng/mL from chitosan/CaSO_4_/PRP microspheres after activation with thrombin [12]. Kutlu et al. showed that scaffolds prepared by the freeze-drying of PRP added to chitosan gel (2% w/v) were more effective than chitosan sponges soaked with PRP on controlled GF release [13]. Oktay et al. applied PRP-embedded chitosan sponges to defects and showed a histological tendency toward increased bone formation [14]. Mathews et al. demonstrated that chitosan enhanced mineralization by upregulating the genes associated with mineralization and calcium-binding proteins [15].

Chitosan is a polysaccharide derived from chitin (copolymers *β*-(1→4)-2-amino-2-deoxy-D-glucose and *β*-(1→4)-2-acetamido 2-deoxy-D-glucose) found in the shells of marine crustaceans and insects and the cell walls of some fungi [16, 17].

In the last ten years, considerable attention has been given to chitosan-based materials in the field of tissue engineering [18, 19]. The beneficial properties of chitosan have been proven, such as biodegradability [20, 21], biocompatibility [22, 23], antibacterial activity [24, 25], cell adhesion [26, 27], and wound healing properties [28, 29]. Moreover, the chemical nature of chitosan gives many possibilities for ionic and covalent modifications that allow for the modulation of the mechanical and biological properties of biomaterials [30, 31].

Regarding the technological aspects, chitosan can be easily processed in diverse forms in the absence of toxic solvents, such as films [32], sponges [33, 34], fibers [35], beads [36], hydrogels [37], and microparticles/nanoparticles [38]. Furthermore, chitosan supports sterilization [39], is abundant in nature, and requires only low-cost processing in nonaggressive ecological conditions before being used as a raw material [17].

Freeze-drying is the most common and simplest method to produce porous chitosan scaffolds. The freezing process provides the nucleation of ice crystals from solution and further growth along the lines of thermal gradients. Exclusion of the chitosan acetate salt from the ice crystal phase and subsequent ice removal by lyophilization generate a porous material [40, 41].

Madihally and Matthew reported that the pore size of chitosan scaffolds produced by lyophilization can be controlled within the range of  1–250 *μ*m [33]. A more uniform and interconnected pore structure can be obtained when lower freezing temperatures are used. Furthermore, the pore orientation is related to the geometry of the moldings used and can also be controlled by changing thermal gradients during freezing [42].

The microstructure, crystallinity, and mechanical strength of the porous chitosan matrix also can be controlled by the chitosan concentration, molecular weight, and deacetylation degree [33, 43].

In this work, the effects of PRP association were studied regarding the porous structure of solid sponges, which were produced by varying chitosan concentration and freezing conditions. The biological performance of association was evaluated in terms of growth factor release kinetics, proliferation, and osteogenic differentiation of seeded human adipose tissue-derived mesenchymal stem cells (h-AdMSCs).

## 2. Experimental

### 2.1. Materials

Chitosan (average molecular weight [Mw] = 4 × 10^5^ Da, degree of deacetylation = 83 ± 4%) was purchased from Polymar (Fortaleza, CE, Brazil) and purified according to the protocol described by Nasti et al. [44]. Other chemicals were of reagent grade and were used without any further purification. All biological experiments were performed with human adipose tissue-derived stem mesenchymal cells (h-AdMSCs) and approved by the Ethics Committee of the Medical Sciences School of the University of Campinas (UNICAMP; CAAE: 0972.0.146.000-11). The donors were healthy individuals aged between 30 and 40 years old who were previously assessed through clinical examinations.

### 2.2. Methods

#### 2.2.1. Preparation of Porous Chitosan Scaffolds (PCHTs)

PCHTs were prepared by freezing at a controlled temperature of a chitosan solution previously poured in 24-well culture plates (TPP, polystyrene, diameter = 15.4 mm) to give them a cylindrical shape, followed by lyophilizing in lyophilizer L101 (Liobras, São Carlos, SP, Brazil) at a temperature of approximately −30°C for 48 hours.

*Effects of Chitosan Concentration*. Chitosan solutions with concentrations of 1, 2, or 3% (w/v) were prepared by dissolution in 0.2 mol L^−1^ acetic acid for 24 hours at room temperature. The solutions were frozen at −20°C for 24 hours and lyophilized. 

*Effects of Freezing Conditions*. Chitosan solution (3% w/v) was prepared as previously described. The solution was frozen at −20°C in a freezer for 24 hours; −80°C in an ultrafreezer for 24 hours; or −196°C by immersion in liquid N_2_; and lyophilized.

#### 2.2.2. Characterization of Porous Chitosan Scaffolds (PCHTs)

*Morphology and Pore Size*. The morphology of PCHTs was evaluated by scanning electron microscopy (SEM) using an LEO 440i Electron Microscopy/Oxford (Cambridge, England) operated at 5 kV accelerating voltage. The scaffolds were gold-coated using a sputter coater POLARON SC7620, VG Microtech (Uckfield, England) for 180 s at a current of 3 mA. Pore size (*n* = 20) was measured using software Image J 1.47t.

*Mechanical Properties.* Mechanical compression tests of PCHTs (*n* = 3) were performed using a Universal Testing Machine, MTS model 810-Flex Test 40 (MTS Systems Corporation, Eden Prairie, MN, USA) up to 60% strain, according to Correia et al. [45]. The testing machine was equipped with a 1.5 kN load cell, and the loading rate was 5 mm/min. Young's modulus was calculated in the initial linear section of the stress-strain curve, when the strain was lower than 10%.

*Degradation in PBS*. The degradation profile of the PCHTs in PBS at 37°C was performed by the gravimetric method described by Tang and Spector through measurements of remaining weight [46]. 

*Water Sorption*. The water sorption was determined by swelling of PCHTs (previously weighted) in PBS (LB Laborclin, Pinhais, PR, Brazil) at pH 7.4 for 24 hours at 37°C. The swollen PCHTs were weighed after the removal of excess water by keeping the surfaces on a filter paper. The swelling ratio (SR) was also calculated using

(1) $$\begin{array}{c} \text{SR}=\frac{ws}{wd}, \end{array}$$

where *ws* and *wd* are the weights of the scaffolds in the swelled state and the dry state, respectively. 

*Porosity*. The porosity (*ε*) of the PCHTs was determined according to the protocol used by Wang et al. and calculated by [47]

(2) $$\begin{array}{c} \varepsilon \left(\%\right)=\frac{Vm-\left(wm/\rho \right)}{Vm}\times 100, \end{array}$$

where *Vm* is the total volume of PCHTs (cm^3^), *ρ* is the density of nonporous CHT (1.342 g/cm^3^), and *wm* is the weight of sponge (g). Values are expressed as the means ± standard deviation (*n* = 3). 

*Cell Compatibility*. The compatibility was carried out by exposing PCHTs to h-AdMSCs followed by cultivation at 37°C for 24 hours and evaluation by MTT assay (3-[4,5-dimethyl-thiazol-2-yl]-2,5-diphenyltetrazolium bromide) (MTT, Molecular Probes) according to a modified Mosmann method [48]. The MTT assay is a colorimetric test based on the reduction of yellow tetrazolium salt into a purple formazan product in presence of cells [49].

#### 2.2.3. PRP Preparations

*PRP Concentration*. P-PRP (plasma rich in platelets and poor in leukocytes) was prepared according to Perez et al. [50]. Briefly, whole blood (WB) was collected into 3.5 mL vacuum tubes (Vacuette, Campinas, SP, Brazil) containing sodium citrate 3.2% (w/v) as an anticoagulant. WB was initially centrifuged in a Rotina 380R centrifuge (Hettich Zentrifugen, Tuttlingen, Germany) at 100 ×g for 10 minutes at 25°C. After the formation of three layers (a bottom layer composed mainly of red blood cells (RBCs); an upper layer composed of plasma, platelets, and some WB cells; and an intermediate layer, or buffy coat, composed mostly of WB cells), only the upper layer was collected to obtain P-PRP. The concentration of platelets, WB cells, and RBCs in WB and P-PRP were determined using the ABX Micros ES 60 hematology analyzer (HORIBA ABX Diagnostics, Montpellier, France).

*P-PRP Activation*. Activated P-PRP (*a*P-PRP) was obtained by adding autologous serum (Ser) and 10% (w/v) CaCl_2_ solution as agonists using the following proportions: agonist/P-PRP = 20% (v/v), Ser/CaCl_2_ volumetric ratio = 9. Autologous serum was prepared by collecting 5 mL of WB in tubes without anticoagulant. After 30 minutes to form clots, WB was centrifuged at 2000 ×g for 10 minutes [51].

#### 2.2.4. The Composite Scaffolds (*a*P-PRP/PCHTs)

*Preparation*. *a*P-PRP/PCHTs was prepared for embedding by dripping *a*P-PRP, immediately after activation, onto PCHTs. The preparation was carried out in 48-well microplates using a ratio of 200 *μ*L of *a*P-PRP to 10–20 mg of PCHTs.

*Release of GFs*. The release of platelet-derived growth factor AB (PDGF-AB) and transforming growth factor *β*1 (TGF-*β*1) was performed after 1 hour of gelation of *a*P-PRP associated with PCHTs in the presence of the culture medium (Dulbecco's Modified Eagle's Medium (DMEM-LG) (Gibco, Grand Island, NY, USA) with low glucose concentration). The culture medium (1.5 mL) was added to *a*P-PRP/PCHTs in 48-well microplates, which were maintained in an incubator with 5% CO_2_ throughout the assays. The total volume of culture medium was withdrawn at 3, 6, 12, 24, and 72 hours, and the same volume of fresh medium was replaced without removing the hydrogels from the wells. The samples were stored at −80°C for further characterization. The concentrations of the released GFs PDGF-AB and TGF-*β*1 were measured using enzyme-linked immunosorbent assay (ELISA) kits (R&D Systems, Minneapolis, MN, USA) according to the manufacturer's instructions and specifications. 

*h-AdMSCs Proliferation*. The cultivation of h-AdMSCs was carried out in 24-well tissue culture plates by adding 1 mL DMEM to the seeded composite scaffolds (*n* = 4). The composite scaffolds seeded were maintained at 37°C for 10 days. Cell proliferation was quantified using the thiazolyl blue tetrazolium bromide (MTT) assay. At 3, 5, 7, and 10 cultivation days, the composite scaffolds were removed and transferred to 24-well plates. MTT (1 mL of 1 mg/mL) was then added, and the cultivation proceeded at 37°C for 4 hours. The MTT solution was then discarded, and 1 mL of DMSO was added to dissolve the purple formazan crystals. The samples were shaken at 120 rpm for 30 min to ensure homogeneous dissolution of the formazan dye, and then 200 *μ*L of each sample was transferred to a 96-well plate. Optical density was measured at 595 nm using a microplate reader (FilterMax F5, Molecular Devices).

The isolation and precultivation of h-AdMSCs as well as the cell seeding were performed as described below. 

*h-AdMSCs Isolation and Precultivation*. Human subcutaneous adipose tissue that was initially acquired from liposuction surgery was washed with sterile PBS, separated into fractions of 10 g, digested with 20 mg of collagenase type 1A and maintained in 20 mL of DMEM-LG containing 10% BSA (bovine serum albumin) and 10 *μ*L of gentamicin for 30 min in a 37°C bath. After complete digestion, the reaction was quenched with 10 mL fetal bovine serum (FBS) and immediately centrifuged for 15 min at 1500 rpm. The supernatant was discarded, and the pellet was suspended in 10 mL DMEM-LG with 10% FBS. After precultivation for 24 h, the culture medium was changed every 3 days; after the fourth passage, the cells were characterized by immunophenotyping (data not shown) using flow cytometry and according to their adipogenic, osteogenic, and chondrogenic differentiation and then used in the subsequent experiments.

*h-AdMSCs-Seeding*. The precultured h-AdMSCs were trypsinized and resuspended in P-PRP to a final cell concentration of 1 × 10^4^ cells/mL. P-PRP containing h-AdMSCs was activated and immediately embedded to the PCHTs in a 24-well tissue culture plate, using 200 *μ*L of h-AdMSCs + *a*P-PRP per 10–20 mg of PCHTs. The composite scaffolds with h-AdMSCs were kept at room temperature for 45 minutes for consolidation of the fibrin network. Pure PRP was used as control.

#### 2.2.5. Images of the h-AdMSCs-Seeded Composite Scaffolds

The images of the cell-seeded composite scaffolds were obtained by scanning electron microscopy after 5 days of h-AdMSCs proliferation. The cell-seeded* composite scaffolds* were fixed in a solution of 4% paraformaldehyde and 2.5% glutaraldehyde in phosphate buffer, pH 7.4, for 2 hours. The samples were than dehydrated in ethanol for 15 min intervals in aqueous 50%, 70%, 95%, and 100% ethanol solutions (2x) and dried at the critical point dryer BAL-TEC CPD 030 (Schalksmühle, Germany). After gold coating (Sputter Coater POLARON, SC7620, VG Microtech), the cell-seeded composite scaffolds were visualized with a SEM (Leo440i, LEO) with an accelerating voltage of 20 kV.

#### 2.2.6. Induction of Osteogenic Differentiation

h-AdMSCs-seeded in the composite scaffolds were induced to differentiate into the osteogenic lineage by providing the osteogenic medium containing DMEM-LG supplemented with 10% FBS, 1% *β*-glycerol-phosphate (Sigma-Aldrich, St. Louis, MO, USA), 1% L-ascorbic acid (Sigma-Aldrich, St. Louis, MO, USA), 1% dexamethasone (Sigma-Aldrich, St. Louis, MO, USA), and 1% penicillin/streptomycin solution (Gibco, Grand Island, NY, USA). The medium was changed every 7 days.

*Evaluation of Differentiation*. Differentiation was evaluated by measuring the alkaline phosphatase activity (ALP) on day 14. The supernatant (200 *μ*L) was collected and mixed with the same volume of p-nitrophenyl phosphate (SIGMAFAST p-nitrophenyl phosphate tablets, Sigma, Saint Louis, MO, USA) as substrate and incubated at room temperature for 30 minutes. Absorbance was read immediately at 405 nm.

#### 2.2.7. Statistical Analysis

Each experiment was carried out in triplicate unless otherwise specified. All results are presented as the mean ± standard deviation (SD). The experimental data from all the studies were analyzed using Analysis of Variance (ANOVA). Statistical significance was set to *P* value ≤ 0.05.

## 3. Results and Discussion

### 3.1. Experimental Design

In this study, we prepared novel composite scaffolds by association of PRP with the 3D-porous structure of chitosan. First, different 3D-porous structures of chitosan (PCHTs) were prepared by freeze-drying by varying the CHT concentration and freezing conditions (temperature and freezing rate). Second, the microstructure and mechanical properties of the PCHTs were characterized and evaluated for cell compatibility. The surface chemistry, hydrophilicity, and positive charge of –NH_2_ groups in acidic medium were maintained unaltered.

The composite scaffolds (*a*P-PRP/PCHTs) were obtained by embedding the PCHTs with immediately activated P-PRP in a 24-well microplate. The release of the GFs (PDGF-AB and TGF-*β*1) from the composite scaffolds was evaluated in DMEM culture medium. Afterwards, precultured h-AdMSCs were added to PRP before activation in order to obtain cell-seeded composite scaffolds.* In vitro* examination of h-AdMSCs proliferation kinetics was performed over 10 days by using the cell-seeded composite scaffolds in DMEM. Additionally, osteogenic differentiation was evaluated by ALP activity measurements after 14 days.

Thus, we verified the correlation between the structure and function of PCHTs by controlling the concentration and freezing conditions of chitosan solution.

### 3.2. Effects of Freezing Conditions and Chitosan Concentration on PCHTs

#### 3.2.1. Images of Porous Structure

**Figure 1:**
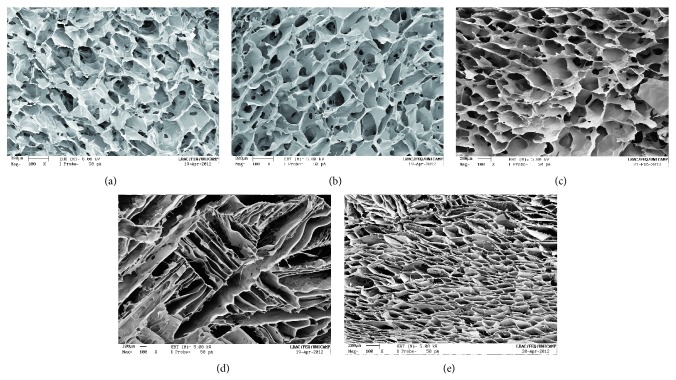
SEM micrographs of PCHTs. Cross-sectional morphologies of (a) PCHTs 1% (−20°C); (b) PCHTs 2% (−20°C); (c) PCHTs 3% (−20°C); (d) PCHTs 3% (−80°C); and (e) PCHTs 3% (−196°C). Original magnification is ×100 and the scale bar represents 200 *μ*m.

Figures 1(a)–1(c) show images of the porous structure from controlled freezing at −20°C by varying the concentration of the chitosan solution (1, 2, or 3%) and subsequent lyophilization. The images show highly porous structures with rounded pores that are uniformly distributed, radially oriented, and visually interconnected, regardless of CHT concentration. Differences in chitosan molecular weight, deacetylation degree, and purity make difficult comparisons even for similar treatment. Madihally and Matthew, showed a higher pore interconnectivity degree and more open pores for 1% (w/w) chitosan concentration for a similar deacetylation degree, but other properties were not characterized [33].

Freezing conditions produced a more prominent effect on the morphology of the pores (Figures 1(d)-1(e)) attributed the variations in the freezing rate used to achieve the temperatures evaluated. In contrast with rounded pores at −20°C (3% CHT) (Figure 1(c)), we observed pores with leaf structure at −80°C (Figure 1(d)), while smaller and elongated open pores were produced at −196°C (Figure 1(e)). In all cases, the pores were uniformly distributed and radially oriented and had a high degree of interconnectivity.

The pore structures allow uniform cell spatial distribution throughout the scaffold, facilitating homogeneous tissue formation. Moreover, the differences in pore morphologies obtained are suitable to several cell linage and type of tissue to be regenerated.

#### 3.2.2. Characterization of PCHTs

*Physicochemical and Mechanical Properties*.  Table 1 shows physicochemical characterization of the PCHTs. At −20°C, the mean diameter of rounded pores could be controlled around the 300–400 *μ*m, regardless of the CHT concentration, although there is a large distribution.

However, the elongated pores from Figures 1(d)-1(e) were markedly decreased with freezing conditions from 2615 *μ*m (−80°C) to 280 *μ*m (−196°C) because crystal growth and hence pore size are functions of both heat and mass transfer rates, determined by the temperature and freezing rate. At −80°C, the observed differences in pore shape and size suggest parallel ice crystal growth, which in turn was caused by the strongly one-dimensional nature of the thermal gradients established during freezing, as discussed by Madihally and Matthew [33].

The diverse nature of tissue architectures requires scaffolds with optimal pore sizes, such as 5 *μ*m for neovascularization [52], 5–15 *μ*m for fibroblast ingrowth [53], 20 *μ*m for the ingrowth of hepatocytes [54], 200–900 *μ*m for osteoconduction [55], and 20–125 *μ*m for regeneration of adult mammalian skin [56].

According to these data, the rounded pores obtained at −20°C or elongated at −196°C are adequate for bone tissue, although other properties could also influence the choice. Moreover, because human adipose tissue-derived mesenchymal stem cells (h-AdMSCs) exhibit a spindle-like shape and are 80–100 *μ*m in diameter and ~200 *μ*m in length [57], the range of pore sizes in our PCHTs allows cells to migrate freely into the scaffolds, favoring the formation of a new tissue.

The porosity is also an important aspect of cell migration and proliferation, guiding and promoting the formation of new tissue. Porosity is defined as the percentage of void space in a solid and is a morphological property independent of material [58]. A porosity higher than 90% and pore interconnectivity are basic requirements for scaffolds in tissue engineering because they affect the diffusion of physiological nutrients and gases to and the removal of metabolic waste and byproducts from cells that have penetrated the scaffold [59, 60]. Moreover, the porosity often compromises the mechanical and structural stability of the scaffolds and must be evaluated in accordance with the application and degradation rate of materials utilized [61].

The porosity values of PCHTs scaffolds ranged from 91.0 to 97.0%, which are adequate for TE, independent of CHT concentration or freezing conditions. However, the porosity decreased with increasing chitosan concentration. The highest porosity was provided from 1% chitosan solution, but there was not a direct relationship with freezing conditions. The PCHTs prepared at −80°C showed a significantly lower porosity even with higher pore size, probably due to their leaf structure (Table 1).

To guide tissue regeneration, scaffolds should also have sufficient mechanical strength during* in vitro* culturing to maintain the spaces required for cell in-growth and matrix elaboration [59]. Moreover, their mechanical properties should be similar to the properties of tissues generated to provide an adequate structural support in the stage of healing [62].

Scaffolds for the regeneration of hard tissue must exhibit a mechanical modulus in the range of 10–1,500 MPa, while scaffolds for soft tissues must exhibit a mechanical modulus in the range of 0.4–350 MPa [63].

In this work, we observed a drastic decrease in Young's modulus (Table 1) with a decrease in CHT concentration. Better mechanical properties were found for a CHT concentration of 3%.

The PCHTs 3% (w/v) prepared at −80°C showed lower mechanical strength than those prepared at −20°C and −196°C with the same CHT concentration, which could be attributed to its leaf structure and pore size. There was no significant difference (*P* < 0.05) in mechanical properties for PCHTs 3% (w/v) frozen at −20°C or −196°C. Thus, the range of Young's moduli found for PCHTs suggests their application for soft tissue engineering. For hard tissues, these scaffolds must be crosslinked and/or reinforced by the addition of fillers.

The water absorption capacity (swelling property) of the scaffolds affects cell growth indirectly [64]. The hydration of the scaffolds is a necessary step for cell incorporation and proliferation.

PCHTs scaffolds showed a high swelling capacity in PBS (pH 7.2) regardless of chitosan concentration and freezing conditions, allowing for rapid hydration when culture medium was added.

*Degradation in PBS*. Scaffold degradation is also an important parameter for the formation of new tissue. The scaffold degradation rate must be tuned appropriately with the growth rate of the new tissue in such a way that by the time the injury site is completely regenerated, the scaffold is completely degraded [55]. Degradation can occur through mechanisms that involve physical or chemical processes and/or biological processes that are mediated by biological agents, such as enzymes [1].

Here, we evaluated* in vitro* the profile degradation of PCHTs in PBS (pH 7.2) at 37°C; we supposed that degradation occurred by solubilization due to the presence of residual acetate molecules.

**Figure 2:**
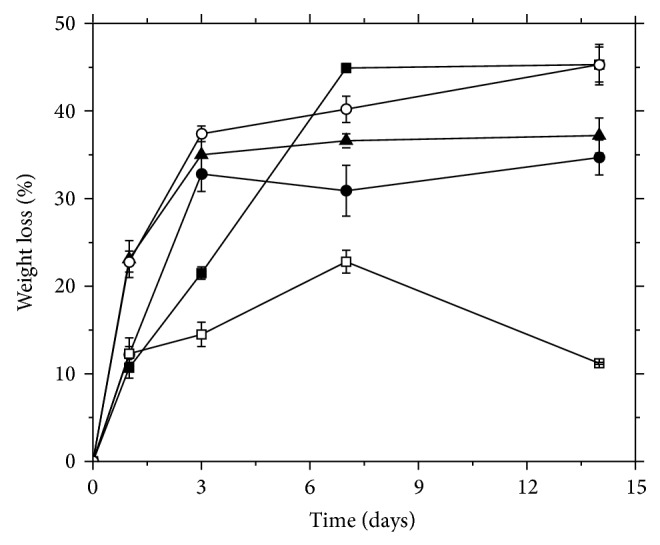
The weight loss of scaffolds with time in PBS at 37°C as a percentage of the original weight of the scaffold (*n* = 3). The data are plotted with the means ± standard error. (■) PCHTs 1% (−20°C); (●) PCHTs 2% (−20°C); (▲) PCHTs 3% (−20°C); (□) PCHTs 3% (−80°C); and (O) PCHTs 3% (−196°C).

Figure 2 shows the degradation profile of PCHTs.

We observed the highest weight loss, approximately 45%, for PCHT 1% (−20°C) after 7 days, while for PCHTs 2% (−20°C), 3% (−20°C), and 3% (−196°C) weight loss was approximately 35% after 3 days. The PCHTs 3% (−80°C) showed the lowest weight loss, probably due to their leaf structure retaining lower concentration of acetate molecules.

Our results suggest that the PCHTs need further stabilization, to ensure the balance between tissue regeneration and degradation rate of the scaffold.

#### 3.2.3. Cell Compatibility

**Figure 3:**
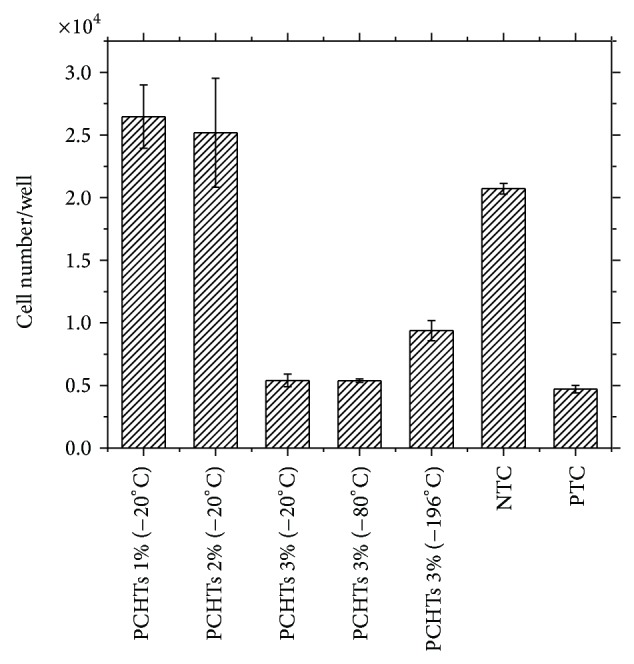
Proliferation of h-AdMSCs exposed to the PCHTs scaffolds after 24 hours of cultivation. Negative control (NTC) = DMEM with 10% FBS; positive control (PTC) = DMEM with phenol 0.5%. Mean ± standard deviation *n* = 3. The population means are significantly different from positive control at ^*^ *P* < 0.05.

Figure 3 shows the cell compatibility of h-AdMSCs cultured in the presence of PCHTs, as assayed by MTT. The results revealed no potential cytotoxicity in 24 hours for the scaffolds according to the standard values (PTC).

However, we observed lower proliferation of h-AdMSCs on PCHTs prepared with chitosan concentration of 3% (w/v) compared to the negative control.

Nevertheless, the PCHTs are potentially useful for* in vivo* applications regardless of CHT concentration and freezing conditions.

### 3.3. Characterization of the Composite Scaffolds

#### 3.3.1. Images of the Cell-Seeded *a*P-PRP/PCHTs

**Figure 4:**
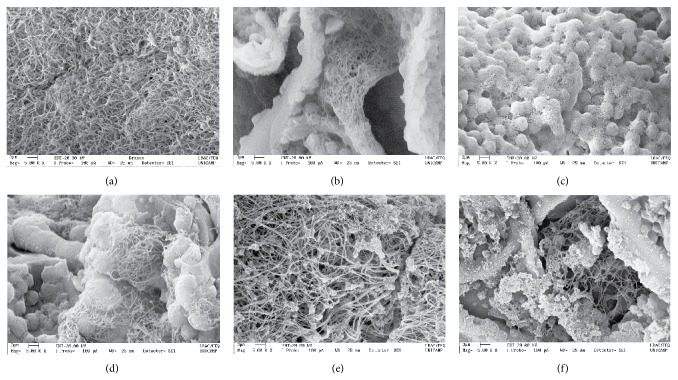
Scanning electron microscopic images of *a*P-PRP/PCHTs after 5 days of cultivation of h-AdMSCs. (a) *a*-PRP; (b) *a*P-PRP/PCHTs 1% (−20°C); (c) *a*P-PRP/PCHTs 2% (−20°C); (d) *a*P-PRP/PCHTs 3% (−20°C); (e) *a*P-PRP/PCHTs 3% (−80°C); and (f) *a*P-PRP/PCHTs 3% (−196°C).

SEM characterization (Figure 4) of the *a*P-PRP/PCHTs on the 5th day of culture showed fibrin networks covering the pores and surface of PCHTs, as a consequence of the interaction of fibrin fibers and CHT. This interaction supported cell proliferation by improving the mechanical strength of the fibrin network, in addition to providing additional surface area to cell adhesion, proliferation, and differentiation.

#### 3.3.2. Growth Factor Release

**Figure 5:**
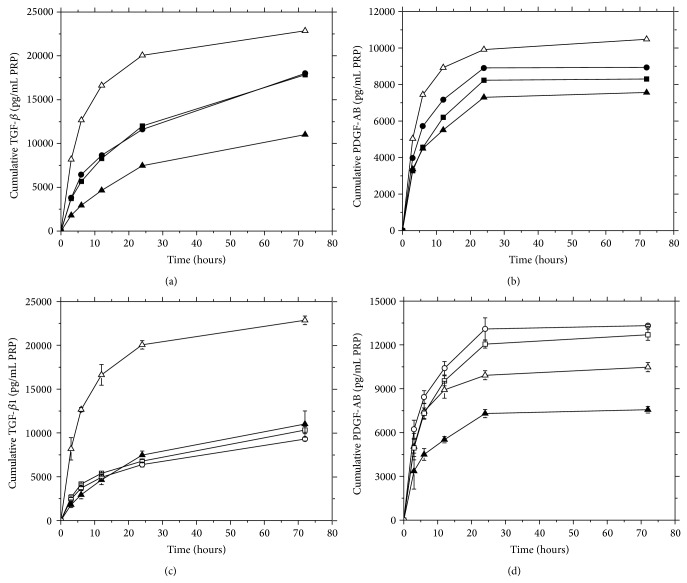
Release profiles of growth factors from *a*P-PRP in porous chitosan scaffolds as a function of ((a), (b)) chitosan concentrations and ((c), (d)) freezing conditions. (■) PCHTs 1% (−20°C); (●) PCHTs 2% (−20°C); (▲) PCHTs 3% (−20°C); (□) PCHTs 3% (−80°C), (O) PCHTs 3% (−196°C), and (∆) P-PRP activated with Ca^+2^/thrombin (used as control); TGF-*β*1 ((a), (c)); and PDGF-AB ((b), (d)). The concentration of platelets in P-PRP was 472,250 pq/mm^3^. Activated P-PRP alone was used as control.

Figure 5 shows PDGF-AB and TGF-*β*1 release kinetics from *a*P-PCHTs determined by ELISA.

The curves show diffusion profiles, indicating no collapse of the porous structure of chitosan scaffolds during the course of the assays, regardless of the CHT concentration and freezing conditions.

PDGF-AB released from PCHTs ended within 24 hours of incubation, whereas TGF-*β*1 tended to continue after 72 hours.

A controlled release of TGF-*β*1 and PDGF-AB was achieved for all CHT concentrations, related to the scaffolds of activated P-PRP only. The slowest release was observed for a chitosan concentration of 3%.

In contrast, for the PCHTs 3% freezing at −80°C and −196°C, we observed a controlled release of TGF-*β*1, but a strong burst release of PDGF-AB.

Thus, PCHTs prepared with 3% CHT at −20°C provided a controlled release of PDGF-AB and TGF-*β*1 and can be an efficient vehicle for release of GFs from PRP.

#### 3.3.3. h-AdMSCs-Seeded Proliferation

**Figure 6:**
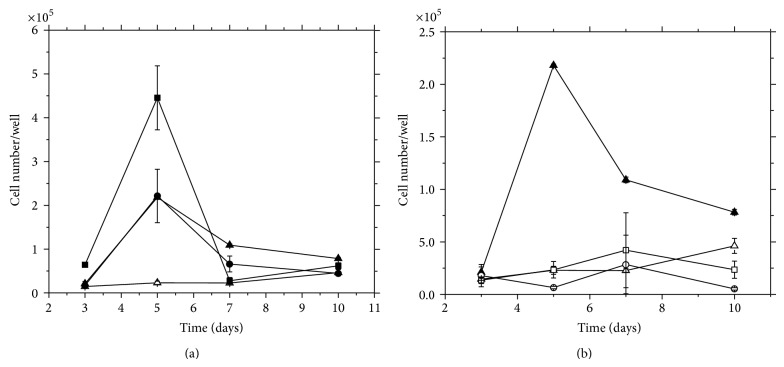
Proliferation kinetic profiles of h-AdMSCs seeded in *a*P-PRP/PCHTs. (a) CHT concentration and (b) freezing conditions. (■) PCHTs 1% (−20°C); (●) PCHTs 2% (−20°C); (▲) PCHTs 3% (−20°C); (□) PCHTs 3% (−80°C), (O) PCHTs 3% (−196°C), and (∆) P-PRP activated with Ca^+2^/thrombin (control). The concentration of platelets in P-PRP was 374,000 pq/mm^3^. Activated P-PRP alone was used as control.

Figure 6 shows the proliferation profile of h-AdMSCs cultured in *a*P-PRP/PCHTs.

The cell number per well determined after 3 days exceeded the number of seeded cells (1.4 × 10^4^ cells/well) in all the *a*P-PRP/PCHTs, meaning that the cells kept in the matrices retained their viability, regardless of the CHT concentration and freezing conditions.

The cell number per well of *a*P-PRP/PCHTs prepared with 1, 2, and 3% (w/v) of chitosan (Figure 6(a)) showed significant differences (*P* < 0.05) compared to control (*a*P-PRP). Therefore, PCHTs should have stabilized fibrin networks as we initially hypothesized.

However, regarding freezing condition, no significant variations for *a*P-PRP/PCHTs prepared at −80°C or −196°C and *a*P-PRP were observed (Figure 6(b)). In all cases, cell viability decreased after 7 days, probably due to collapse of the structure.

We also observed that the exponential phase of the cultures (Figure 6(a)) started after the maximum release of GFs (Figure 5), and the decline phase matched the largest weight loss of the PCHTs (Figure 2).

#### 3.3.4. Induction of Osteogenic Differentiation

**Figure 7:**
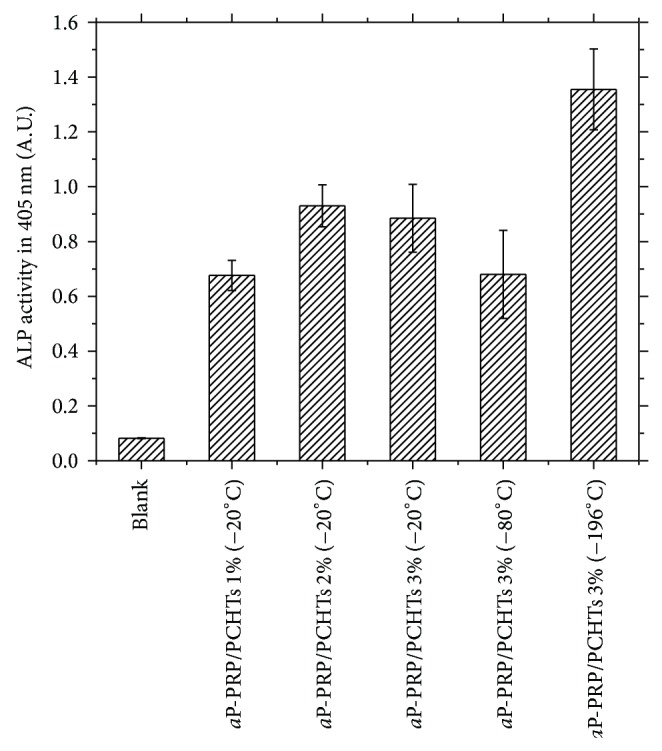
ALP activities of cells cultured on *a*P-PRP/PCHTs scaffolds prepared with different CHT concentrations and freezing conditions (statistically significant differences from blank, *n* = 3, ^*^ *P* < 0.05). Blank = the reagents used in the assay only. The concentration of platelets in whole blood donors (average of 2 donors) was 163,500 pq/mm^3^. After preparation of the PRP, the platelets were concentrated approximately 1.74 times, with an average final concentration of 303,000 pq/mm^3^.

Osteogenic differentiation of h-AdMSCs was investigated 14 days after cell seeding. ALP, an early marker of osteogenic differentiation, was determined, and the results are shown in Figure 7. The expression of ALP activity showed no significant difference (*P* < 0.05) with CHT concentration. However, higher osteogenic differentiation was obtained with *a*P-PRP/PCHTs 3% (−196°C).

## 4. Conclusions

Composite scaffolds were prepared with porous chitosan and *a*-PRP. The performance of the composite scaffolds was superior to *a*P-PRP alone, indicating that the porous chitosan stabilized the fibrin network, supporting our initial hypothesis.

Chitosan concentration and freezing conditions influenced the physicochemical and biological properties of the scaffolds. On the average, physicochemical, mechanical, and h-AdMSCs proliferation improved by the use of 3% (w/v) chitosan and −20°C freezing temperature, while −196°C favored osteogenic differentiation. Additional stabilization of the porous structure is needed for applications in regenerative medicine.

## Figures and Tables

**Table 1:** Physicochemical characterization of PCHTs.

CHT concentration (%)	Freezing temperature (°C)	Swelling ratio (*n* = 3)	Pore size (*µ*m) (*n* = 20)	Porosity (%) (*n* = 3)	Young's moduli (MPa) (*n* = 3)
1	−20	6.5 ± 0.4	425 ± 102	97.1 ± 0.3	0.037 ± 0.002
2	−20	5.5 ± 0.9	418 ± 113	94.7 ± 0.8	0.5 ± 0.1
3	−20	4.7 ± 0.9	336 ± 138	93.1 ± 0.5	1.1 ± 0.1
3	−80	5.1 ± 0.9	2615 ± 600^*^	91.7 ± 0.3	0.28 ± 0.01
3	−196	6.4 ± 0.6	280 ± 62^*^	92.5 ± 0.1	1.01 ± 0.05

^*^Mean longitudinal size.
